# Acceptability and Feasibility of Survivorship Group Medical Visits for Breast Cancer Survivors in a Safety Net Hospital

**DOI:** 10.1007/s13187-024-02429-6

**Published:** 2024-05-10

**Authors:** Evelin Trejo, Ana I. Velazquez, Elizabeth Castillo, Paul Couey, Barbara Cicerelli, Robin McBride, Nancy J. Burke, Niharika Dixit

**Affiliations:** 1 Division of Hematology/Oncology, Department of Medicine, University of California San Francisco, San Francisco, CA USA; 2 Zuckerberg San Francisco General Hospital and Trauma Center, San Francisco, CA USA; 3 Helen Diller Family Comprehensive Cancer Center, University of California, San Francisco, CA USA; 4 Department of Public Health, University of California Merced, Merced, CA USA

**Keywords:** Cancer survivorship, Breast cancer, Group medical visits, Safety net, Patient navigation

## Abstract

Providing cost-effective, comprehensive survivorship care remains a significant challenge. Breast cancer survivors (BCS) who have limited income and are from marginalized racial and ethnic groups experience a worse quality of life and report higher distress. Thus, innovative care models are required to address the needs of BCS in low resource settings. Group medical visits (GMV), utilized in chronic disease management, are an excellent model for education and building skills. This single-arm intervention study was conducted at a public hospital in California. GMVs consisted of five 2-h weekly sessions focused on survivorship care planning, side effects of treatment and prevention, emotional health, sexual health, physical activity, and diet. The patient navigators recruited three consecutive GMV groups of six English-speaking BCS (*N* = 17). A multidisciplinary team delivered GMVs, and a patient navigator facilitated all the sessions. We used attendance rates, pre- and post-surveys, and debriefing interviews to assess the feasibility and acceptability of the intervention. We enrolled 18 BCS. One participant dropped out before the intervention started, 17 BCS consistently attended and actively participated in the GMV, and 76% (13) attended all planned sessions. Participants rated GMVs in the post-survey and shared their support for GMVs in debriefing interviews. The BCS who completed the post-survey reported that GMVs increased their awareness, confidence, and knowledge of survivorship care. GMVs were explicitly designed to address unmet needs for services necessary for survivorship care but not readily available in safety net settings. Our pilot data suggest that patient-navigator-facilitated GMVs are a feasible and acceptable model for integrating survivorship care in public hospitals.

## Introduction

Cancer survivors report a high rate of unmet needs [1–4]. Breast cancer survivors (BCS), who are racial and ethnic minorities and low-income, experience worse health-related quality of life outcomes following cancer treatment [5, 6]. Cancer and its treatment can significantly impact BCS’ physical and emotional health; once the treatment ends, many cancer survivors feel abandoned by their oncology team and express concerns that their primary care physicians (PCP) lack the expertise to provide cancer survivorship care [7, 8]. Due to the limited time during oncology and primary care visits, discussions about long-term side effects and the overall impact of cancer treatment on patients’ emotional and sexual health are generally only briefly addressed [9, 10]. Previous studies have shown that handing patients a survivorship care plan has a limited impact on cancer survivors’ quality of life [11]. In an earlier study, Napoles et al. described the needs of cancer survivors, including (1) symptom management, (2) psychosocial support, (3) support to address feelings of abandonment by the healthcare system, and (4) information about healthy lifestyles [12]. Napoles et al. proposed that a planned intervention must include skill development for symptom management, stress management, communication with providers, family, and friends, information about symptoms and side effects, follow-up care, signs of recurrence, community resources, healthy lifestyles, and social support to address distress and improve health-related quality of life (HRQOL) [12]. Finally, survivorship care is multidisciplinary, and cancer survivors require access to additional services like physical therapy, nutrition, and psychologists [13, 14].

BCS from marginalized communities face multiple barriers to accessing survivorship care. In addition to race and ethnicity, social determinants of health, such as financial well-being, insurance type, and education attainment, influence the HRQOL of cancer survivors [15]. For example, Black/African American breast cancer survivors are more likely to experience symptoms related to endocrine therapy, which can contribute to lower adherence to treatment [16]. Most BCS who have limited income, are racial and ethnic minorities and receive care in public health systems lack access to and underutilize survivorship care resources even when these resources are available [17, 18]. In addition, they are more likely to experience racial discrimination [15, 19]. Latina BCS experience worse HRQOL and higher levels of pain, fatigue, and depressive symptoms as compared to White women [5, 12] Furthermore, they are less likely to receive survivorship care services such as survivorship care information [20, 21] and more likely to report unmet symptom needs [22]. While Latina BCS are more likely to experience anxiety and fear of cancer recurrence [12, 23], they are less likely to receive psychosocial health services [23].

The Group medical visits (GMV) model is an excellent model for patient education and building skills, and it has been utilized in chronic disease management, specifically to support healthy lifestyle interventions and build patient skills [24, 25]. There is extensive literature outlining the benefits of GMV, including improvement in clinical outcomes such as hemoglobin A1C (for DM patients) [24], improved access to care [26], patient empowerment, and reduction in stress and loneliness. Also, GMV accommodates longer appointments and social interactions for patients while providing a more efficient, lower cost, and more sustainable healthcare intervention than usual care from the perspectives of providers and the healthcare systems [26–31]. GMV structure allows for longer appointments with dedicated time for discussing BCS concerns. Furthermore, GMV may provide access to services rarely available to BCS in a resource-limited setting. GMV model is not as widely utilized in oncology. There are only small studies ranging from follow-up for women with BRCA [32] chemotherapy education [33] or addressing concerns related to a single discipline such as gynecological care, [34] smoking cessation, [35] mindfulness, [28] and integrative oncology services [26]. Finally, some small pilots of multidisciplinary care of cancer survivors [36–40] have been reported. These studies only provide preliminary evidence of improved patient experience [36–39], with one study providing evidence for lower fat consumption [36] and another providing evidence for a reduction in the number of visits to the specialists [40]. However, there are no studies of GMV addressing the needs of marginalized BCS who receive care in public hospital settings that serve individuals with limited income, also called safety net hospitals in the USA. 

To address the unmet needs of cancer survivors and harness the unrealized potential of GMV in the multidisciplinary care of cancer survivors, we created and implemented a 5-session survivorship Group medical visit (GMV) intervention entitled Survive4life tailored to low-income breast cancer survivors receiving care in a safety net hospital. The GMV was implemented by a multidisciplinary team, including an oncology provider, patient navigators, clinical psychologists, sexual health counselors, and PCP. The curriculum covers survivorship care planning, long-term side effects, and emotional and sexual health. Each session focused on the survivors’ current concerns. Additionally, consultants with expertise in specific topics delivered the educational content and provided resources.

## Methods

### Study Setting

We conducted a single-arm pilot mixed methods study to determine the feasibility and acceptability of a patient-navigator-facilitated GMV intervention in breast cancer survivors (BCS) in a public hospital serving low-income residents. Healthcare institutions serving low-income, uninsured, and underinsured immigrants are called safety-net institutions in the USA. Most patients served by the institution are uninsured or on public health insurance for low-income individuals in the USA, known as Medicaid. The barriers to care at the institutions are low health literacy, limited English proficiency, housing and food insecurity, and transportation barriers. There are limited resources for supportive care and survivorship care services at the institutional level, including limited availability of services such as behavioral health, nutrition services, physical therapy, and sexual health counselors; however, the institution houses a robust patient navigation program for women with breast cancer and BCS. The navigators play an important role in increasing access to treatment for BCS [41].

### Eligibility and Recruitment

The university institutional review board approved this study. Eligibility criteria included BCS within 1–5 years of completing active breast cancer treatment, including surgery, chemotherapy, and radiation, the ability to read and write in English, and the ability to provide consent. Patients with h/o breast cancer who were receiving endocrine therapy were eligible to participate. Patients with breast cancer undergoing active treatment such as radiation or chemotherapy with a diagnosis of metastatic breast cancer were excluded. The study coordinator screened prospective participants and confirmed the eligibility criteria. Patient navigators introduced the study to the prospective participants and gave a flyer to them. The study coordinator invited participants who expressed interest to participate. The participants demonstrated their understanding of the study procedures and signed consent forms.

### Intervention

Building on prior work on the role of patient navigation in cancer survivorship care and the needs of breast cancer survivors in safety net settings [42], we developed a GMV intervention with the help of a team that included patient navigators, oncologists (MD/Advanced practice provider (APP)), nurses, dietician, clinical psychologist, and sexual health counselor. An experienced patient navigator served as the facilitator of the GMV session. The GMV intervention included five 2-h patient-navigator-facilitated sessions delivered weekly. The sessions were designed to (1) introduce a survivorship care plan (SCP)(2) emphasize post-treatment care, symptom management, and the importance of primary care; (3) provide BCS tools to manage emotional health, and (4) educate BCS on sexual health and provide a safe and supportive space for BCS to discuss sexual health issues. The session structure included blood pressure and weight measurements, and dedicated time for questions and answers. BCS also had an individual check-in with the oncology APP. Individual check-ins were 5–10 min per person and were designed to review individual survivorship care plans in the first session and individual concerns in the subsequent sessions (Table 1). All sessions ended with a goal-setting homework informed by motivational interviewing. At the following session, the participants shared their success with the homework.

**Table 1:** Survive4Life intervention

Week	Topic	Description
Week 1	Introduction	Welcome to the participants Introduction to GMV, expectations for participation Introduction to Survivorship Care Plan (SCP) Role of SCPs in cancer care: Information about treatment received—surveillance for recurrence of cancer, delayed and long-term side effects, health maintenance, including information about screening for other cancers, diet, and physical activity Questions and answers session Homework: Encourage participants to discuss SCP with their oncologists
Week 2	Long-term effects of treatment	Welcome and check-in and follow up from the last session Discussion with oncologists post-treatment care including surveillance for cancer, symptom management, staying healthy, and the importance of primary care Questions and answers Homework: Encourage participants to make appointments with their primary care physicians
Week 3	Emotional health	One-on-one check-in Follow up from prior session about primary care appointment Emotional health challenges in cancer survivorship Tools to manage emotional health and fear of cancer recurrence Questions and answers Homework: encourage participants to practice tips on management of stress
Week 4	Sexual health	One-on-one check-in Follow up on the management of stress and if participants practiced the tips from the previous session Educate BCS on sexual health and provide a safe and supportive space for BCS to discuss sexual health issues Questions and answers Homework: To do a self-care activity
Week 5	Celebration of survivorship	One-on-one check-in Follow up on self-care activity from the prior session Follow up about all prior sessions Informal social gathering and celebration of survivorship

Over a period of 1 year, we enrolled three BCS cohorts, each with 6–7 participants. The following five sessions were offered: (1) introduction to survivorship care plans, (2) long-term effects of treatment and taking control of health, (3) emotional health, (4) sexual health, and (5) celebration of survivorship (Table 1). An oncology provider discussed survivorship care plans which included details of treatment, surveillance for recurrence, long-term and delayed side effects of treatment, and health maintenance. A PCP joined the second session with an oncologist and addressed the role of primary care in cancer survivorship which included health maintenance, management of co-morbidities, screening for other cancers, and role of primary care in symptoms management and referral to services such as behavioral health. In the third session, a clinical psychologist discussed the emotional concerns; these concerns specifically include managing stress and anxiety and fear of cancer recurrence. In the fourth group session, the sexual health counselor discussed sexual health, focusing on self-care and managing symptoms, such as vaginal dryness, atrophy, low libido, and intimacy after cancer. The fifth session included a follow-up from prior sessions. The session included a reminder to the BCS of how far they have come, a brief review of all they have learned, and a celebration of survivorship, with an informal social gathering without a structured education component.

### Role of Patient Navigators

Patient navigators had a significant role in the development of the intervention. They introduced the study to the eligible participants, who were then approached by the study coordinator for recruitment. The patient navigators have expertise in health education and motivational interviewing, which they leveraged as facilitators. A patient navigator facilitated each session. The patient navigator introduced the GMV intervention, established ground rules, and introduced each expert to the group. Patient navigators also encouraged participants to ask questions, encouraged group sharing, and facilitated discussions between the participants and the educator. Patient navigators provided clarification where needed and encouraged participants to ask questions. Patient navigators also supported the BCS after the session in facilitating referrals to community resources or contacting their primary care physicians.

### Data Collection and Analysis

We collected demographics at enrollment. We conducted pre- and post-surveys to evaluate the intervention. As the focus was more on recruitment, retention, and implementation, the pre- and post-surveys were voluntary. Participants also filled out a pre-intervention NCCN distress thermometer, which was offered routinely to patients with cancer at the institution for psychosocial distress screening [1, 43]. Finally, we conducted debriefing conversational interviews with participants to understand their experience of the intervention.

All participants received transportation support and a $25 gift card for each session for up to $100. In addition, the patients who participated in the debriefing interviews received a $40 gift card. We used Microsoft Excel (Microsoft Corporation. Seattle, USA) for data collection and analysis. We generated descriptive statistics and used the Fisher exact test of significance for categorical variables with a *P*-value of 0.05 as significant.

### Feasibility and Acceptability

We examined the feasibility by evaluating the following: (1) feasibility of recruitment of planned sample size (enrollment > 70%), (2) Delivery of the intervention as designed (> 80%), (3) representativeness of the sample of breast cancer survivors in our practice, (4) collection of pre- and post-intervention surveys (> 70%). We examined acceptability by intervention completion rate (> 70%), evaluation of the intervention with the post-survey, and follow-up conversational interviews with the participants.

### Qualitative Conversational Interviews

We invited nine participants from three GMV cohorts who consented to be interviewed within 2 months of the four GMV series between mid-2019 and early 2020. We recorded the interviews, transcribed them using NVivo transcription software, and crosschecked the accuracy of the audio recording. We destroyed the audio recordings after the transcription was completed. ET and NB conducted interviews. We analyzed the interviews using a priori theme and added new codes. ET and ND independently coded the interviews. Finally, the codes were mapped onto themes relevant to acceptability and feasibility.

## Results

We invited 21 BCS, and three declined to participate; we recruited 18 participants, and one participant dropped out after recruitment due to time conflict. Table 2 details the participants’ demographic profiles. The median age of the participants was 51–60. Of 17 participants, six reported race as Asian, five were African American, three were non-Hispanic white, and two were Hispanic. All participants had public health insurance.

**Table 2:** Participant demographic profile

Characteristics	*n* (%)
Age	
20–30	0 (0)
31–40	2 (11)
41–50	3 (17)
51–60	4 (22)
61–70	6 (33)
71 years or older	2 (11)
Gender	
Male	0 (0)
Female	17 (94)
Race/ethnicity	
Asian/Pacific Islander	6 (35)
African American/Black	5 (28)
Hispanic	2 (11)
White/Caucasian	3 (17)
Other	1 (6)

### Acceptability and Feasibility

Recruitment was feasible, with 18 of 21 invited agreeing to participate with a response rate of 85%; however, one dropped out with a sample size of 17 for a final response rate of 80%. All sessions were delivered as planned with a 15/15 (100%) completion rate. The diversity of the sample represents the safety net institution’s English-speaking population of BCS. Approximately 50% of breast cancer survivors receiving care in our institution speak English; other common languages are Spanish and Chinese (Cantonese). Pre- and post-survey completion were 17 (100%) and 13 (76%), respectively. Intervention completion rates were high, with all participants attending at least one session of their cohort and 13(76%) attending all sessions in their respective cohorts.

#### Implementation Process

All sessions were delivered as planned, with minimal changes. An example of modification is that we prioritized question-and-answer sessions based on the first cohort to make the sessions more interactive in subsequent cohorts. All participants engaged with the medical team and asked questions during the sessions.

#### Evaluation of Intervention

All participants completed the pre-survey, and 13 completed the post-survey. Additionally, 10 participants also completed the NCCN Distress Thermometer. In the post-survey, 67% of BCS strongly agreed that they were familiar with their survivorship care plan compared to the pre-survey 22% (*P* 0.002), 67% of BCS strongly agreed that they were confident in recognizing signs and symptoms of the long-term side effects of breast cancer as compared to 28% (*P* 0.002) in the pre-survey, and 67% of BCS strongly agreed in the post-survey that they were aware of community programs and services that might benefit them as a cancer survivor, compared to 33% (*P* 0.010) in the pre-survey (Table 3). On a scale of 1–5, with a score of one signifying not useful and five as very useful, 78% reported that the information provided was helpful for PCP/Oncology and Sexual Health sessions. Seventy-two percent found the emotional health session very helpful (Table 4).

**Table 3:** Participant confidence in survivorship care

Perspective	Measure of view	Pre-survey	Post-survey
		N 17 (100%)	N 13 (100%)
I am familiar with my Survivorship Care Plan	Strongly agree/agree Disagree/strongly disagree	10 (55) 7 (45)	13 (100)
I am confident in recognizing signs and symptoms of the long-term side effects of breast cancer	Strongly agree/agree Disagree Strongly disagree	14 (78) 3 (22)	13 (100%)
I am aware of the community programs and services that might benefit me as a cancer survivor	Strongly agree agree Disagree/strongly disagree	10 (61) 7 (39)	13 (100)
I am confident in communicating information regarding my cancer treatment with my primary care provider	Strongly agree/agree Disagree Strongly disagree	17 (100)	13 (100)

**Table 4:** Participants’ evaluation of Group medical visit sessions

Information	Scale (1 = not useful, 5 = very useful)	*n* (%)
How useful was the information on PCP/Oncology	1 2 3 4 5	0 (0) 0 (0) 0 (0) 0 (0) 14 (78)
How useful was the information on Sexual Health	1 2 3 4 5	0 (0) 0 (0) 0 (0) 0 (0) 14 (78)
How useful was the information on Emotional Health	1 2 3 4 5	0 (0) 0 (0) 1 (6) 0 (0) 13 (72)

### Preliminary Data on NCCN Distress Thermometer

We invited all participants to complete an adapted NCCN distress thermometer of which ten participants completed a pre-intervention NCCN distress thermometer. On a scale of 0–10, with zero measuring no distress and 10 indicating extreme distress, participants were asked how much distress they felt that day and in the past week. Six participants reported moderate or severe distress (4 or more), and three reported mild distress. Among participants, money was identified as a practical problem (33%). Feeling tired was the most commonly reported physical symptom (39%), followed by tingling in hands and feet (28%), and weight changes (33%). Emotional problems included worry (28%), fear (23%), and loss of interest in the usual activities (23%).

### Conversational Interviews

Nine conversational interviews were conducted with GMV participants to understand their experiences with GMV. Analysis of these interviews resulted in the identification of three major themes related to the GMV intervention. These include group cohesion, group structure, and the role of patient navigators. We provide explanations of these themes along with subthemes with illustrative quotes as follows:

### Group Cohesion

#### Shared Experiences Increased the Sense of Peer Support in the Group

Participants reflected on their shared experiences of cancer treatment and managing side effects, such as hot flashes, which made them feel understood and supported. They described a sense of support and of strength in being part of the group.

When I talk to the people in the group, we have hair falling and hot flashes, they know exactly what I mean. It’s kind of nice and we can support each other and we can be kind to each other, we get stronger, and especially a survivor group, that means everyone has passed that already.

And then going to the groups, it helped me not feel like I was alone. That was a big piece of my recovery because I actually stepped outside of the box for a minute to reach out to see if there was anything there for me to hold on to.

#### Group Participation Helped with a Sense of Isolation

Many BCS report a sense of isolation after completion of treatment as they are expected to resume their role in the family and may not have any family support. Furthermore, BCS may not always be able to share their survivorship experiences, fear and anxiety, with their close family and friends. Sharing their experience with other survivors in the group allowed the BCS to feel less isolated and well-supported. GMV provided them with a safe and supportive environment to share these concerns and, in turn, feel more in control of their life. One participant reported.

I didn’t have nobody at the time that I was going through this except for my roommate and my team, ‘cause I live in alone. I don’t have family here. My family didn’t find out until way later. But I’m just saying that it felt-it made me feel supported.

In addition, being part of the GMV also helped BCS feel more in control of their own life.

The support group…Being a part of it made me own it and made me take shape of my life and direct it.

#### Group Structure

We sought feedback from the BCS regarding the structure of the GMV sessions. The participants reflected on the small group size as supportive environment that fostered sharing.

#### Breast Cancer Specific Small Group

The participants appreciated being part of a peer GMV explicitly designed for women diagnosed with breast cancer. Participants who had attended other support groups in the past that had both men and women and other kinds of cancer sites where the participants did not feel comfortable. The participants reported that the small size of GMV and focus on breast cancer with patients who had completed treatment and were on surveillance made them feel more comfortable.

I went to that group but it was like 35 people, I counted once and then there were guys there, men who had other cancers so there were different kinds of cancers. And I kind of didn’t relate as well and I don’t know, if it was right for me. And this group was all breast cancer and we all were still in treatment or not kind of treatment or through treatments just under surveillance just to make sure that none returns.

Participants who are introverted may sometimes feel uncomfortable sharing their thoughts and experiences in a group setting. However, the small group format and focus on shared experiences made the GMV format non-intimidating. They felt at ease attending and sharing in this format. One participant even shared that despite being an introvert, she felt comfortable in the group setting and attended the final session, which was an informal social gathering focused on follow-up and celebrating survivorship, rather than a structured session with a speaker.

I’m serious, ‘cause I’m a really isolated type of person. I’m really introverted. I stay to myself. It doesn’t bother me. But to be in a room of other people that shared my experience, it meant something to me. I even showed up to the party.

#### Collective Learning

According to one participant, the question-and-answer format of GMV was an essential element. Other participants often raised questions that they themselves may not have thought to ask, thus enhancing the collective learning experience of the group.

Cause there’s certain questions that don’t come to mind at that second, but if there’s other peers around you, they come up with, question the then you’re kind of like, oh yeah, that was the question that I wanted to ask, and you get that information that way too.

#### Access to the Medical Team

During the session, participants emphasized the importance of having the oncology team (APP or MD) included in the GMV team. This gave them the freedom to ask the oncology team questions, independent of the session speaker, thus providing greater access to their medical team. This contrasts with a support group setting where patients do not have access to their medical team.

Because every session they have someone like ______ to be there to answer all the questions. If they have some questions to ask, then they can get the answer straight away, so that’s nice.

#### Easily Understandable Content

In a safety net setting, it is crucial to tailor information to make it easily accessible to participants. The participants shared their feedback on the sessions’ content, noting that they particularly appreciated how all of the medical information shared was at a level they could easily understand highlighting the need for tailoring content.

The material is presented to you, but it’s not presented on a medical level. It’s presented at everyday level that you could understand.

### Role of Patient Navigators

#### Patient Navigators as Facilitators Created a Supportive Environment

The patient navigators who acted as facilitators played a crucial role in creating a safe and supportive environment for breast cancer survivors (BCS) who were hesitant to speak up and engage. The navigators provide logistical, social, and emotional support during treatment and survivorship, which helped put the BCS at ease. Their presence as facilitators and support staff throughout the duration of GMV made the BCS feel comfortable attending and sharing their experiences.

I went there the first time I was quiet, because I was observing what’s going on, but at least I see navigators and some people that I know already, so it’s not too bad. And then I went in and I met with ______ and other people who you know. Everybody’s nice, and we laugh a lot, and we share our experience. At the end of the day, I see, it’s nice to be there, and listen to them, and feel like “Oh, they have the same experience.”

#### Patient Navigators Have Expertise to Help Cancer Survivors

Since the navigation program provides referrals to services in addition to the emotional support and addressing barriers to care, they have a unique role in the delivery of survivorship care. One participant suggested that navigators themselves should lead a session that includes information about community resources. This would enable the BCS to learn about the services available and how to access them, as well as provide the navigators with an opportunity to share their knowledge and expertise.

Maybe some of the navigators or social worker can come in for one session and talk about the sources for us. So I know we get some sources from the navigator, but if somebody can come and talk about that, sometimes it may be better for us too.

## Discussion

Our study found that patient-navigator-facilitated GMV was both feasible and acceptable in a safety-net hospital setting. Pre- and post-surveys showed that GMV led to increased patient confidence in their knowledge of survivorship care plans and their ability to manage the late and long-term effects of cancer. Qualitative data revealed that BCS appreciated the GMV format delivered by their oncology team, which allowed them ample time to have their questions answered. Participants reported feeling supported by their peers and the medical team. These findings are consistent with prior research in other chronic diseases, where the GMV model has been frequently utilized [25, 28, 44–46].

We found that GMV intervention can play a critical role in addressing the varied and complex needs of cancer survivors. Along with medical care, such interventions can be designed to address psychosocial and informational needs. While Survivorship care plans have been suggested as a way to support cancer survivors’ informational needs, it is worth noting that these plans can be time-consuming to develop and implement [47], and the implementation of survivorship care plans has been patchy. Furthermore, survivorship care plans have not been shown to improve patient care outcomes [11]. In our prior work [42], we found that survivorship care plans in a safety net setting did not improve quality of life or self-efficacy, and survivorship care must be provided separately from routine surveillance.

GMVs also harness the power of supportive peer interactions and the lived experience of BCS and provide a forum for BCS to have their questions answered in a supportive and receptive environment. Finally, GMVs focus on cancer survivorship needs other than surveillance.

Our intervention was specifically designed to address the unmet need for multidisciplinary services necessary for cancer survivorship. While there are a few small studies on GMV in managing cancer survivorship care issues [34, 37–39], our work adds unique knowledge in this field by addressing the needs of low-income BCS who receive care in a public hospital in a low-resource setting. We leveraged an existing patient navigation program to deliver the GMV rather than the medical team taking the lead to provide an intervention focused on survivorship. It is well known that patient navigators can improve communication and patient empowerment [41], and we found that patient navigators’ presence made the participants feel more comfortable. Providing cost-effective, comprehensive survivorship care remains a significant challenge for which optimal delivery models are needed. Our work shows that GMV can be included in current survivorship care models to provide multidisciplinary services to cancer survivors. Finally, although we designed this intervention to be sustainable and allow billing for services in the USA, we did not specifically look at this outcome in this study [48].

This study has limitations. Since our participants were drawn from a single health center, generalizability to other care settings is limited. However, the principles of survivorship on which the GMV was based are universal. Participants were not required to answer every survey. Thus, we are limited by the information that respondents chose not to disclose because the survey was voluntary. We are specifically limited by lower rates of completion of post-survey and NCCN distress thermometer. Finally, this was a small sample size and thus any efficacy consideration are only hypothesis-generating and need to be confirmed in larger studies. Finally, our study was limited to BCS who spoke English and thus may not be applicable to BCS who have limited English proficiency.

## Conclusions

GMVs were designed to address the multidisciplinary unmet needs of BCS. Our participants reported high satisfaction with survivorship education, emotional and social support, and health management support received in GMV. Finally, larger studies are needed to assess other outcomes related to survivorship care, such as adherence to cancer surveillance, health-related quality of life, adherence to non-cancer-related care, and psychosocial outcomes, such as fear of cancer recurrence.
